# Simulating autosomal genotypes with realistic linkage disequilibrium and a spiked-in genetic effect

**DOI:** 10.1186/s12859-017-2004-2

**Published:** 2018-01-02

**Authors:** M. Shi, D. M. Umbach, A. S. Wise, C. R. Weinberg

**Affiliations:** Biostatistics and Computational Biology Branch, National Institute of Environmental Health Sciences, Research Triangle Park, Durham, NC USA

**Keywords:** Genotype simulation, Genome-wide association, Case-parent triads, Linkage disequilibrium, Epistasis

## Abstract

**Background:**

To evaluate statistical methods for genome-wide genetic analyses, one needs to be able to simulate realistic genotypes. We here describe a method, applicable to a broad range of association study designs, that can simulate autosome-wide single-nucleotide polymorphism data with realistic linkage disequilibrium and with spiked in, user-specified, single or multi-SNP causal effects.

**Results:**

Our construction uses existing genome-wide association data from unrelated case-parent triads, augmented by including a hypothetical complement triad for each triad (same parents but with a hypothetical offspring who carries the non-transmitted parental alleles). We assign offspring qualitative or quantitative traits probabilistically through a specified risk model and show that our approach destroys the risk signals from the original data. Our method can simulate genetically homogeneous or stratified populations and can simulate case-parents studies, case-control studies, case-only studies, or studies of quantitative traits. We show that allele frequencies and linkage disequilibrium structure in the original genome-wide association sample are preserved in the simulated data. We have implemented our method in an R package (TriadSim) which is freely available at the comprehensive R archive network.

**Conclusion:**

We have proposed a method for simulating genome-wide SNP data with realistic linkage disequilibrium. Our method will be useful for developing statistical methods for studying genetic associations, including higher order effects like epistasis and gene by environment interactions.

**Electronic supplementary material:**

The online version of this article (10.1186/s12859-017-2004-2) contains supplementary material, which is available to authorized users.

## Background

Evaluation of new statistical methods typically requires simulations. Generating realistic genotype simulations at a genome-wide scale remains challenging, however. Ideally, simulation methods should produce realistic allele frequency and linkage disequilibrium (LD) profiles while allowing investigators to spike in (and then try to find) multi-SNP causal effects against a null background. The genetic simulation tools currently available take different approaches to simulation and offer different capabilities; the National Cancer Institute has provided a web resource that catalogues existing software packages and aids comparisons of their characteristics (https://popmodels.cancercontrol.cancer.gov/gsr/). Most current methods for simulating extensive genome-wide data mimic evolutionary processes, either forward in time (e.g., [1–3]) or backward in time through coalescent theory (e.g., [4, 5]). Such approaches are well suited for addressing population-genetics questions; and, although they can be applied to generate pseudo-samples for evaluating statistical methods, setting needed and influential simulation parameters appropriately can be challenging for those not expert in evolutionary genetics. Resampling existing data is another approach to generating genome-wide simulations (e.g., [6, 7]). Provided suitable data are available, resampling approaches are conceptually straightforward and generally successful at retaining allele frequencies and LD structure from the source data; but they are more restricted in some applications than approaches that mimic evolution.

The many available genetic simulators differ widely in their features and ease of use. We sought an approach that was conceptually straightforward and would deliver realistic LD structure. Those considerations led us toward a resampling-based approach. We sought an approach that would simulate genotype data for case-parents designs and for case-control designs. In addition, we wanted to be able to model traits flexibly -- either dichotomous or quantitative phenotypes -- and be able to include possible epistatic interactions and gene-environment interactions as contributing to phenotypes. No available simulator seemed to achieve all of those goals simultaneously.

We propose a resampling-based simulation method that can generate genome-wide autosomal SNP genotypes under various risk scenarios. Our method requires existing autosomal genotype data from a genome-wide association study (GWAS) of case-parent triads as a starting point and largely preserves the allele frequencies and LD structure in that data. It creates simulated case-parents data by resampling genotype fragments sequentially from different families and concatenating them. Trait phenotypes, either dichotomous or quantitative, are then assigned to offspring at random based on a user-specified risk model. Though the method is applicable to multiple SNPs that act independently, we focus on risk models that involve one or more sets of interacting SNPs (to be referred to as “pathways”) with or without gene-environment interactions. If the available GWAS data contains identified subpopulations, the method can simulate either a homogeneous or a stratified population. Though the construction uses case-parents data, simulated samples from other study designs are achieved by retaining subsets of the simulated genotypes (e.g., discarding the simulated parents); for example, population-based random samples for quantitative traits (with or without parents) and case-control samples are possible.

We begin by briefly outlining some features of our R package followed by presenting our re-sampling algorithm for case-parents data and describing how we assign trait values to simulated offspring. We then document the performance of our approach with several simulations. We close with a brief discussion.

## Implementation

Our method is implemented in an R package called “TriadSim” (https://cran.r-project.org/web/packages/TriadSim/index.html). The input files for the package are triad genotype data in the widely-used PLINK format. The output files are also in PLINK format. The user can nominate a single SNP or multiple SNPs in “pathways” (sets of SNP loci) through the input parameter “target.snp”. Alternatively, the user can specify a desired allele frequency for the SNPs in each pathway, the number of pathways and the number of SNPs in each pathway and allow the program to pick the SNPs in the pathways. The program allows for an array of user-specified parameters such as the number of simulated subjects, the number of break points to be used for each chromosome, exposure prevalence and the baseline disease prevalence among noncarriers. The input parameters also include a few Boolean variables to allow the user to perform simulations for different types of outcome: “qtl” for designating a quantitative trait rather than a dichotomous trait; “is.case” for simulating a case-triad rather than a control-triad. The user also needs to input risk parameters that quantify the effect of the genotype(s) on the trait. Statistical models for case-parents data estimate relative risks (RR), e.g. equation (1), whereas the logistic models for case-control data estimate odds ratios (OR). For a rare disease, OR and RR are numerically similar; but for a common disease, their ratio depends on the disease prevalence. Accordingly, our package allows users to input either relative risk or odds ratios with an indicator variable “is.or” to denote whether odds ratios are the input. The program can take advantage of a multi-core computer by running multiple processes in parallel.

## Results

### Algorithm

#### Resampling to generate null data

**Fig. 1:**
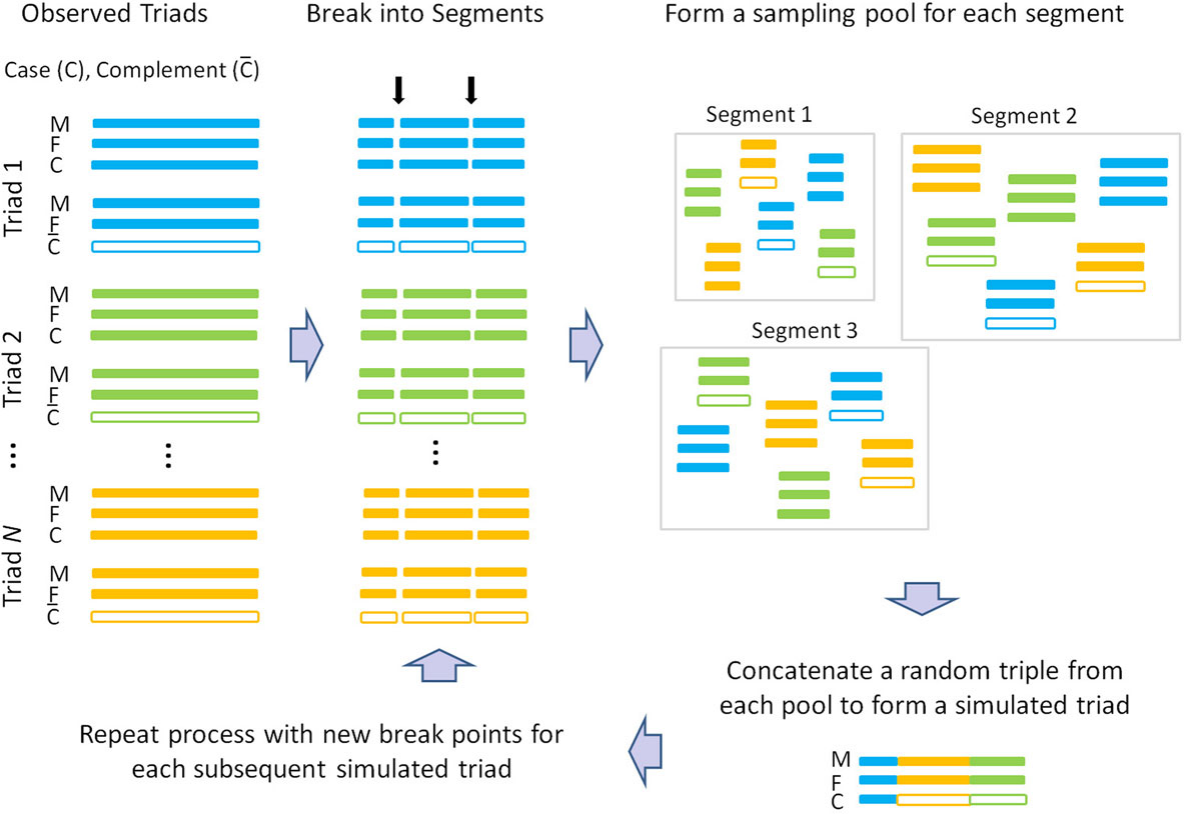
A schematic drawing of the resampling procedure. Triads from three different families are shown in different colors. The solid bars represent the original GWAS subjects and open bars represent their corresponding complements. The triads used by our resampling algorithm include both case and complement triads. Break points are introduced at random and kept the same for the mother, father, and child genotypes across the mix of all observed and complement triads. Each chromosome is broken into several fragments with a mother-father-child triplicate fragment from a given chromosomal location treated as a unit. For each sequential location along the chromosome, one forms a location-specific fragment pool consisting of all the triplicate fragments from that location. A simulated triad is created by randomly sampling a triplicate fragment from each location-specific fragment pool in turn and then sequentially splicing the sampled fragments to make simulated chromosomes. The entire process of creating location-specific fragment pools is repeated for each subsequent simulated triad, starting with a new random set of breakpoints so that every simulated triad is based on a distinct fragmentation pattern

For input, our algorithm requires actual GWAS data from a case-parents study: genotypes of an affected offspring and the two biological parents. We assume the data have been subjected to some quality control so that, for example, triads with evident nonpaternity or an adopted offspring have been excluded. As depicted in Fig. 1, we augment the GWAS data with a hypothetical complement triad for each observed triad; the complement triad has the same parental genotypes but its offspring carries the parental alleles not transmitted to the case. We then randomly select, for each chromosome, a fixed number of break points (we used three) at recombination hotspots and keep these break points the same across the three individuals in each triad and across all triads to be sampled to create a given simulated triad. (To ensure genetic diversity, the break points are selected anew for each simulated triad in turn.) Breaking the chromosomes in this way creates a collection of mother-father-child triples for each chromosomal fragment, one from each case or complement triad. We construct each simulated triad genotype by resampling a triple at random with replacement from the collection for each chromosomal fragment and concatenating them sequentially (Fig. 1). By treating such triples as the resampling units, we preserve realistic LD structure and transmission patterns and do not impose any random-mating assumption. The inclusion of the complement triads serves to destroy any risk signals in the original GWAS data. We then also randomly switch labels for the mother and the father in order to remove potential asymmetries due to maternally-mediated genetic effects or asymmetric mating in the original data.

#### Assigning trait phenotypes associated with sets of SNPs

The algorithm as described to this point generates triads under a global null. To simulate under alternative hypotheses, trait phenotypes are assigned probabilistically according to a specified trait model. One can generate either dichotomous or quantitative phenotypes. A trait model provides a stochastic rule for assigning an individual offspring genotype to a particular trait value. For dichotomous traits like the presence of a disease, the trait model is a risk model that specifies the offspring’s probability of being affected conditional on genotype; disease status is assigned at random based on that probability. For quantitative traits, the trait model typically specifies the offspring’s expected trait value; adding a randomly-generated perturbation assigns the trait value.

For simplicity, all the trait models that we consider have as predictors some function of the offspring’s genotype. The function is a linear combination of *p* indicator variables, denoted ***β***^′^***X*** where ***β*** = (*β*_1_, *β*_2_, …, *β*_*p*_)^′^ is a vector of parameters and ***X*** = (*X*_1_, *X*_2_, …, *X*_*p*_)^′^ is a vector of indicator variables. An indicator variable can be simple; for example, an indicator that the subject carries one or more copies of the variant at a particular SNP locus. Thus, ***X*** might encode indicators for *p* distinct SNPs that each contribute to the trait outcome. Our focus, however, is on epistatic scenarios where the risk is increased by inheritance of a particular combination of variant alleles in one or in multiple pathways. The indicator variables are then the product of a set of SNP-specific indicator variables. For example, a scenario may involve two pathways (*p* = 2), a 4-SNP and a disjoint 3-SNP pathway. Then, *X*_1_ would be the indicator that the subject carries at least one variant allele at each of the four loci in pathway 1, *X*_2_ would be the indicator that the subject carries at least one variant allele at each of the three loci in pathway 2, and *β*_1_ and *β*_2_ would assess the magnitude of each pathway’s influence on the trait. One can use the same software to generate simulations where risk depends on single SNPs by regarding them as 1-SNP pathways.

For a dichotomous disease phenotype, we model the penetrance among those with vector ***X*** as:

1 $$ \log \left(P\left( Affected|\boldsymbol{X}\right)\right)=\alpha +{\boldsymbol{\beta}}^{\hbox{'}}\boldsymbol{X} $$

Here, *α* is the log risk of disease among individuals who do not have a complete set of SNPs for any single pathway. As described above, each component of ***X*** is a product of locus-specific indicator variables and, for dichotomous traits, ***β*** is a vector of the log relative risks for the associated pathways. If two or more pathways are present in one individual, the model shown in (1) implies that their contributions combine multiplicatively on the relative risk scale. For case-parents triad data, only families with affected offspring are retained in the final data set. For case-only data, the user discards the parents. For control-parents data, only families with unaffected offspring are retained. For case-control data, the algorithm retains affected and unaffected offspring according to a user-specified ratio and the user discards parental genotypes.

For a quantitative trait, we model the trait value as:

2 $$ \left(Y|\boldsymbol{X}\right)=\alpha +{\boldsymbol{\beta}}^{\hbox{'}}\boldsymbol{X}+\epsilon $$

Here *Y* denotes a quantitative trait with a mean of *α* among noncarriers. Again, each component of ***X*** is a product of SNP-specific indicator variables, ***β*** is their corresponding vector of pathway-specific shifts of the mean, and *ϵ* is a normally distributed mean-zero random error term. With two or more pathways involved, we assume that their effects are additive on the original scale. The algorithm retains all offspring, regardless of trait value; though our software returns parental genotypes, they can be discarded subsequently.

For scenarios involving gene-environmental interactions, we consider only a dichotomous exposure, denoted *E*, coded as 1 for present and 0 for absent. For dichotomous traits, we model penetrance as follows:

3 $$ \log \left(P\left( Affected|\boldsymbol{X},E\right)\right)=\alpha +{\boldsymbol{\beta}}^{\hbox{'}}\boldsymbol{X}+\theta E+{\boldsymbol{\gamma}}^{\hbox{'}}E\boldsymbol{X} $$

Here *α* the log risk of the disease among the unexposed individuals who do not have a complete set of SNPs for any single pathway. ***β*** is a vector of the log relative risks for the associated pathways in unexposed individuals. *θ* is the log relative risk associated with exposure among individuals who do not have a complete set of SNPs for any single pathway (exposure main effect) and ***γ*** = ( *γ*_1_, *γ*_2_, …, *γ*_*p*_) is a vector of the log interaction effects. The corresponding model for quantitative traits can be expressed similarly by including the terms for the exposure main effect and the interaction in formula (2).

#### Accommodating population structure

Provided the input GWAS data contain more than one identifiable genetically distinct sub-population (e.g., ethnicity), our implementation also allows for the simulation of a stratified population by sampling separately from GWAS data specific to each sub-population. Each sub-population has its own allele frequency distribution implicitly from the input data. In addition, the user specifies, separately for each subpopulation, its proportion in the underlying population targeted by the simulation, exposure prevalence (if relevant), and disease prevalence or mean trait value among (unexposed) non-carriers (we assume that other risk parameters are common across sub-populations). To simulate a setting where there would be bias due to population stratification, one should select alleles for the risk model that differ in frequency between the two identified sub-populations. Sub-population-specific disease prevalence or mean trait values are achieved by setting the *α* parameter to different values in each sub-population. Our program randomly selects a sub-population from which to generate a simulated triad with probability given by the desired underlying sub-population proportions, then it simulates the offspring and parent genotypes and determines the offspring phenotype probabilistically as described above. The program loops through these steps until it accumulates the targeted number of retained triads (case, control, or quantitative trait).

### Evaluating genetic characteristics of simulated data sets

To evaluate the performance of our software, we conducted simulations using the cleft consortium GWAS data downloaded from dbGaP as the input genotype source (International Consortium to Identify Genes and Interactions Controlling Oral Clefts, Accession number: phs000094.v1.p1). These data included complete triad genotypes for 1899 families in two identified ethnic groups, 1028 Asian and 871 Caucasian. For these simulations, we set the number of break points at three for each chromosome.

#### Elimination of existing risk signals

The original cleft GWAS had identified several risk loci for facial clefts [8]. We verified that our resampling algorithm destroys the risk signals present in the original data, by first simulating data under the null scenario of no risk-increasing SNPs. For simplicity, we simulated data for 10,279 loci on four chromosomes; we chose chromosomes that contained the clefting risk loci that had been reported with *p* < 5 × 10^−8^ (chromosomes 1, 8, 17, and 20). We used triad families of Asian and Caucasian origins in homogeneous and stratified scenarios. For homogeneous scenarios, all simulated triads are from just one ethic group; we provide results for Asian and Caucasian families separately. For stratified scenarios, we used both the Caucasian and Asian triads as the source population. The underlying proportion of the Caucasian population was set as 0.46 and the ratio of baseline disease prevalences was set as 1.3 (Caucasian to Asian). For each null scenario, we generated 2000 null data sets, each containing 1000 triads, a number close to the sample sizes of the two subpopulations in the original cleft study. Signals from the 14 loci reported at genome-wide significance level by the original GWAS study were all successfully obliterated in the simulated data as indicated by Type I error rates near the nominal per-comparison α-level of 0.05 when testing those loci for associations with risk (Table 1).

**Table 1:** Original genetic signals (indicated by *p* values) are absent in the simulated data

SNP	Original GWAS^a^			Type I error rates using simulated data ^b^		
	Asian	Caucasian	Both	Asian	Caucasian	Both
	*n* = 1028	*n* = 871	*n* = 1899	*n* = 1000	*n* = 1000	*n* = 1000
rs560426	3.84E-08	1.73E-03	1.12E-09	0.063	0.044	0.045
rs481931	6.93E-05	1.22E-03	3.04E-07	0.054	0.041	0.049
rs4147811	3.08E-05	6.16E-04	6.99E-08	0.057	0.043	0.053
rs2073485	1.24E-07	5.93E-01	4.02E-06	0.054	0.043	0.050
rs2013162	7.98E-07	2.98E-01	1.02E-05	0.052	0.038	0.061
rs861020	1.38E-04	7.34E-03	4.01E-06	0.055	0.047	0.056
rs10863790	7.31E-09	1.14E-01	2.01E-09	0.048	0.045	0.049
rs987525	8.53E-04	2.94E-12	1.74E-14	0.042	0.054	0.051
rs6072081	1.90E-06	2.10E-03	2.52E-08	0.045	0.053	0.040
rs6065259	1.00E-05	1.19E-02	7.57E-07	0.055	0.047	0.048
rs17820943	1.50E-07	5.70E-03	9.81E-09	0.038	0.059	0.051
rs13041247	8.80E-08	4.56E-03	4.92E-09	0.040	0.058	0.048
rs11696257	9.39E-08	5.07E-03	5.88E-09	0.041	0.057	0.053
rs6102085	8.67E-08	1.23E-01	5.00E-07	0.046	0.055	0.050

^a^The p values were based on the complete triads, which were used in the simulation study

^b^Based on a per-comparison α-level of 0.05 and 2000 simulated studies

#### Preservation of LD structure and minor allele frequencies

**Fig. 2:**
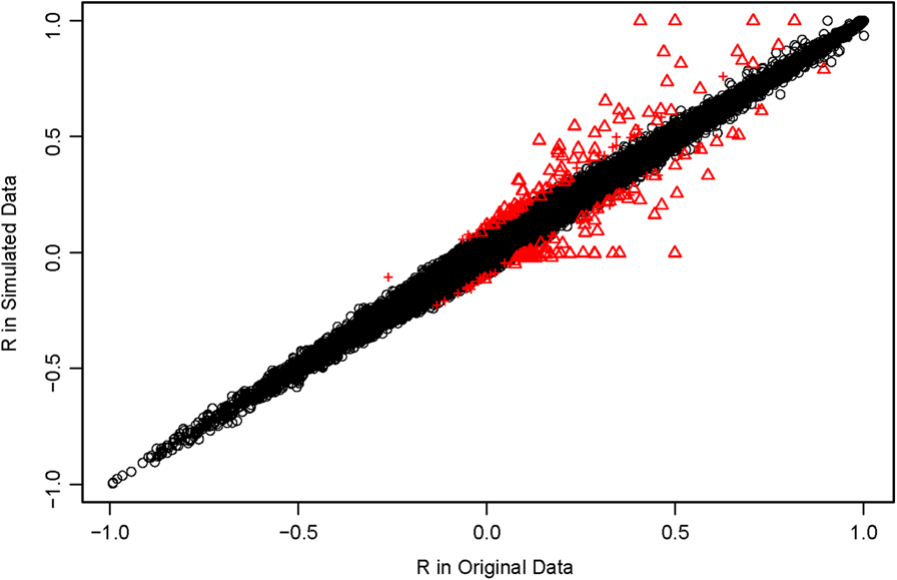
Genotype correlation (R) between loci within 200Kb of each other in the original data plotted against the corresponding R in a single simulated data set. Red crosses represent the SNP pairs with an observed R that differs from that based on the original data by at least 0.1 and where the MAF is at least 0.02 in both SNPs in the original data (0.1% of the correlations shown are in this category). The red triangles represent the SNPs pairs with a correlation coefficient differing from the original data by at least 0.1 and where the MAF is less than 0.02 in either SNP in the original data (0.3% of the correlations shown are in this category)

Simulated null data based on the Asian subpopulation also provided evidence that our algorithm preserves the original LD structure in the genome. For pairs of SNPs within 200 kb of each other, we compared the pairwise SNP correlations between the original data and the simulated data. LD (as assessed by the correlation coefficient based on genotypes (0, 1, 2)) between pairs of SNPs in the original data was well preserved in the simulated data. Among all SNP pairs, the correlation between pairwise LD measured in the original data and the average pairwise LD across 1000 simulated null data sets was 1.00. On average across 1000 simulated data sets, the absolute difference between correlations was less than 0.1 for 99.6% of SNP pairs. Among the exceptions, about 71% on average involved SNP pairs with low minor allele frequencies (MAF) (MAF <0.02, red triangles in Fig. 2) for which LD may change simply because of sampling variation for the rare allele frequencies. Examining pairs of rare SNPs (MAF ≤ 0.05) more closely, we found that those LD discrepancies between the original and the simulated samples that exceeded the 0.1 threshold appeared most often when the MAF for both SNPs was <0.005 (Additional file 1: Fig. S1).

**Fig. 3:**
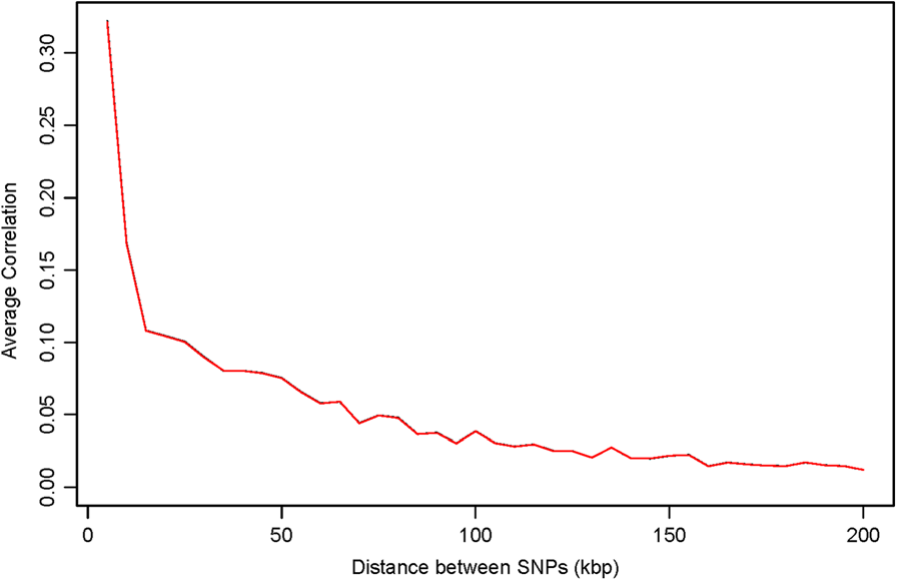
Average squared genotype correlations (R^2^) between loci plotted against the distance between them. The black line shows the curve based on the original data while the red line shows the corresponding averaged value based on 1000 simulated data sets. The two lines coincide and only the red line is visible

The decay in average pairwise LD with increasing inter-SNP distance was similar in the original and simulated data (Fig. 3). When restricted to SNPs with MAF ≤ 0.05, the matching between the decay with inter-SNP distance curves for the original and simulated data is less perfect, as indicated by the minor separation of the red and black trajectories in Additional file 1: Fig. S3 compared to Additional file 1: Fig. S2; the more jagged appearance in Additional file 1: Fig. S3 is attributable to limited numbers of rare SNP pairs.

**Fig. 4:**
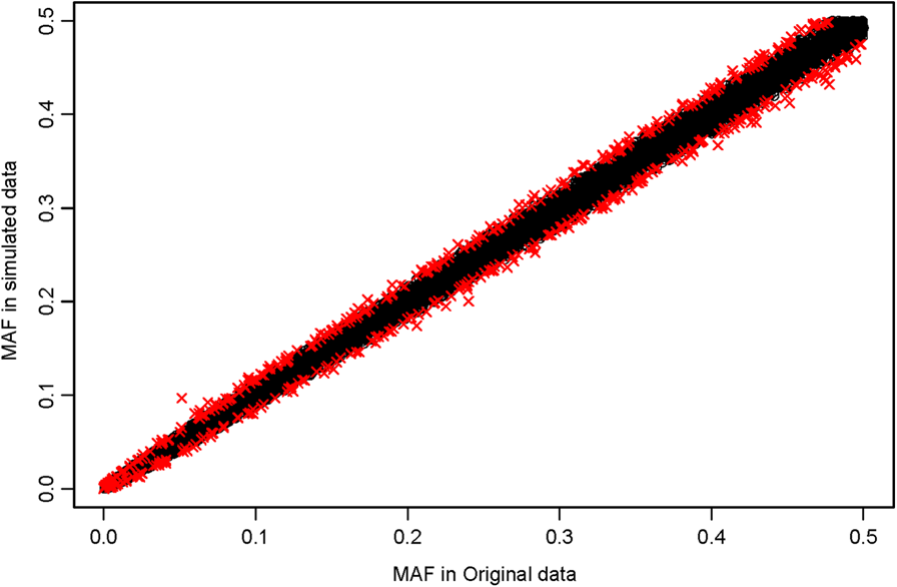
Comparison of minor allele frequencies (MAFs) in the original data versus those in a single simulated data set. The red crosses represent the SNPs with MAF in the simulated data that fall outside 95% binomial prediction intervals calculated using the SNP’s MAF in the original data as its true MAF (4.9% of the SNPs are in this category)

**Fig. 5:**
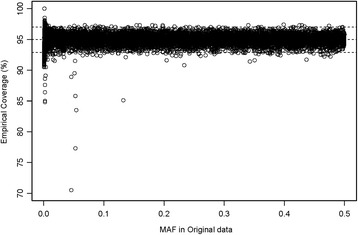
Empirical coverage of nominal 95% binomial prediction intervals plotted against the SNP’s minor allele frequency (MAF) in the original data. Prediction intervals are calculated for each SNP in each simulated data set using the SNP’s MAF in the original data as its true MAF. Empirical coverage for a SNP is calculated as the proportion of 1000 simulated data sets in which the SNP’s observed MAF was within its prediction interval. Each point represents empirical coverage for one of 10,279 SNPs in the simulations. The horizontal reference lines correspond to mean and median coverage across all SNPs (both 95% coverage, matching the nominal coverage) and to the 2.5th and 97.5th percentiles (93% and 97% coverage, respectively), One SNP at MAF = 0.051and with coverage less than 70% does not appear in the figure

In addition to preserving LD, our simulation method preserved the original allele frequencies, the correlation between the MAF in the original data and the average MAF for the same locus across the 1000 simulated null data sets approached 1 (Fig. 4 shows an example based on a single simulated data set). On average across the 1000 data sets, the absolute difference in allele frequencies was less than 0.02 for 96.9% of the SNPs. Regarding a SNP’s MAF in the original data as its true MAF, we calculated 95% binomial prediction intervals for the MAF observed for each SNP in a new simulated sample. In a typical sample, ~95% of the SNP-specific MAFs from the simulated sample fell within those prediction limits, including rare SNPs (Fig. 4; Additional file 1: Fig. S4). Across 1000 simulated data sets, the empirical coverage of the SNP-specific prediction intervals (i.e., the proportion of simulations in which a SNP’s simulated MAF fell within its 95% prediction limits) had both median and mean 95% across all SNPs (Fig. 5). Those values were relatively constant across all true MAFs, though the mean coverage fell slightly and variability in coverage increased for rare SNPs (MAF ≤ 0.005) (Fig. 5 and Additional file 1: Fig. S5).

We conclude that our simulation procedure provides simulated data that successfully mimics both the LD structure and the minor allele frequencies present in the original input data, though with some minor degradation among rare alleles.

#### Proper insertion of SNP-associated traits

We also simulated data under different scenarios to verify that the trait-related pathways that we spiked in could be recovered analytically. For all these simulated scenarios, we used the same 10,279 loci and assumed two causative pathways, each with four interacting loci. We selected the eight pathway SNPs for these simulations from among SNPs with the targeted allele frequencies and selected SNPs that were widely spread across four chromosomes. First, we studied stratified null scenarios using 2000 simulated studies of 1000 triads each. We wanted to create stratification that would generate substantial bias under a naive analysis; consequently, we needed two subpopulations that differed in both allele prevalence and baseline disease rate. For these null scenarios, the two subpopulations were separately resampled from the Asian and Caucasian GWAS data, respectively. We selected SNPs to ensure that the allele frequency of each of the SNPs in the two pathways designated for testing was close to 0.15 and 0.5 in the two subpopulations, respectively. The underlying proportion of the second population was set at 0.46 (mimicking Caucasian proportion in the clefting data). The baseline disease risks in the two subpopulations were set to 0.17% and 0.5% for a dichotomous trait (Table 2, Stratified null) and the shift in mean was set to 1.1 for a continuous trait with standard deviation 1 (Table 3, Stratified null). For each alternative scenario, we simulated 1000 data sets, each with 1000 triads from a homogeneous population based on the Asian subpopulation. We simulated data under scenarios with pathway genetic effects only and scenarios with gene-environment interactions. Frequencies for the individual SNPs at each locus in the pathways were around 0.2 or 0.3. We selected these values for MAFs because they were typical of the single SNPs detected as risk-associated in the clefting GWAS and values for relative risks that were likely to give reasonably tight confidence limits for a study with 1000 triads. Assessing performance over a range of allele frequencies is outside the scope of this paper. For a dichotomous trait, the baseline risk of the disease was set at 1.66 per 1000 individuals and the relative risks associated with carrying at least one variant allele at all SNPs in the pathway were 1.65 and 2.71 for the two pathways, respectively (Table 2, Alternative). For gene-environment interactions, we considered a pure-interaction scenario where the relative risks for each pathway’s genetic main effects and for the exposure main effect were set at 1 while the interaction effects were set at 1.65 and 2.71 for the two pathways, respectively (Table 4, Dichotomous). For the quantitative trait, the trait mean was set at zero and the mean shifts for those carrying at least one variant alleles at all SNPs in the pathway were 0.1 and 0.15 for the two pathways, respectively (Table 3, Alternative). For gene-environment interactions, we retained 0.1 and 0.15 as the genetic main effect parameters, set the exposure main effect parameter to 0, and had the interaction induce a 0.02 greater shift in mean for the exposed (Table 4, Continuous). To analyze these data, we fit the same trait model used to generate them; in other words, we sought to demonstrate that the estimated parameters tracked the true parameters assuming that we knew the true pathways in advance.

**Table 2:** Analytic recovery of pathway genetic effects for a dichotomous trait, based on 1000 (under alternatives) or 2000 (under the null) simulated studies of 1000 triads

Simulation Setup				Simulation Results			
Scenario	Allele Frequency for each of the 4 SNPs in each pathway	True Relative Risk		Average Estimated Relative Risk (95% CI for the mean)		Estimated Coverage of Nominal 95% CIs	
		Pathway 1	Pathway 2	Pathway 1	Pathway 2	Pathway 1	Pathway 2
Stratified null	a	1	1	1.00 (0.99,1.01)	1.00 (0.99,1.01)	0.948	0.952
Alternative ^b^	0.3	1.65	2.71	1.65 (1.63,1.66)	2.73 (2.71,2.75)	0.948	0.959
Alternative ^b^	0.2	1.65	2.71	1.64 (1.61,1.67)	2.72 (2.67,2.77)	0.963	0.964

^a^By design, the allele frequency for each of the 4 SNPs in each tested pathway in subpopulation one was close to 0.15 while that in subpopulation two was close to 0.5

^b^Simulations under alternatives used homogeneous populations

**Table 3:** Analytic recovery of pathway genetic effects for a continuous trait, based on 1000 (under alternatives) or 2000 (under the null) simulated studies of 1000 offspring

Simulation Setup				Simulation Results			
		True Shift in mean		Average Estimated Shift in Mean (95% CI for the Mean Shift in Mean)		Estimated Coverage of Nominal 95% CIs	
Scenario	Allele Frequency for each of the 4 SNPs in each pathway	Pathway 1	Pathway 2	Pathway 1	Pathway 2	Pathway 1	Pathway 2
Stratified null	a	0	0	0.00 (−0.02,0.03)	−0.01 (−0.03,0.02)	0.949	0.958
Alternative ^b^	0.3	0.1	0.15	0.10 (0.09,0.11)	0.15 (0.14,0.15)	0.949	0.95
Alternative ^b^	0.2	0.1	0.15	0.10 (0.09,0.12)	0.14 (0.13,0.16)	0.949	0.96

^a^By design, the allele frequency for each of the 4 SNPs in each pathway in subpopulation one was close to 0.15 while that in subpopulation two was close to 0.5

^b^Simulations under alternatives used homogeneous populations

**Table 4:** Analytic recovery of gene-environment interaction effects for dichotomous and continuous traits in a homogeneous population, based on 1000 simulated studies of 1000 triads (dichotomous) or offspring (continuous)

Phenotype	Allele Frequency	Pathway	Truth or Estimate	Parameter Values^a^		Estimated Coverage of Nominal 95% CIs	
				Pathway Genetic Effect	GxE Interaction Effect	Pathway Genetic Effect	GxE Interaction Effect
Dichotomous	0.3	1	True	1	1.65		
			Estimated	1.00 (0.99,1.01)	1.65 (1.61,1.69)	0.953	0.954
		2	True	1	2.71		
			Estimated	1.00 (0.99,1.02)	2.72 (2.67,2.77)	0.936	0.954
Dichotomous	0.2	1	True	1	1.65		
			Estimated	1.01 (0.98,1.04)	1.79 (1.67,1.91)	0.959	0.954
		2	True	1	2.71		
			Estimated	1.01 (0.98,1.04)	3.05 (2.80,3.33)	0.958	0.957
Continuous	0.3	1	True	0.1	0.02		
			Estimated	0.10 (0.09,0.11)	0.02 (0.00,0.04)	0.944	0.966
		2	True	0.15	0.02		
			Estimated	0.15 (0.14,0.16)	0.02 (0.00,0.03)	0.948	0.941
Continuous	0.2	1	True	0.1	0.02		
			Estimated	0.10 (0.08,0.11)	0.03 (−0.01,0.07)	0.945	0.949
		2	True	0.15	0.02		
			Estimated	0.17 (0.15,0.18)	0.02 (−0.02,0.06)	0.936	0.933

^a^Parameter values relate to relative risks for dichotomous traits and to mean shifts for quantitative traits. For all models, we assumed that in the absence of either genetic pathway there was no effect of the dichotomous exposure

For a dichotomous trait, we estimated the pathway genetic risk parameters in both the null and alternative scenarios without bias (Table 2); in addition, empirical confidence interval coverage agreed well with the nominal 95%. For a quantitative trait, we also estimated the pathway genetic shift parameters in both the null and alternative scenarios without bias (Table 3), and empirical confidence interval coverage matched the nominal 95%. We saw the same unbiased-estimation and confidence-interval-coverage properties when the scenarios included gene-environment interactions (Table 4). We conclude that our approach to spiking multi-SNP causal effects is operating properly.

#### Processing time

We assessed the computation time based on a multi-processor computer with AMD Opteron Processor 6380 with a CPU speed 1400 MHz and 504 G memory. We ran our program with 5 parallel processes. For each simulated data set of 1000 triads, the program took under three minutes for 10 k SNPs but took about 35 min for ~500,000 SNPs (Table 5). The main time-limiting step seems to be file read and write rather than the resampling step based on the risk model since the time difference is minor for diseases with different prevalences, especially when the number of SNPs is large.

**Table 5:** Simulation time for generation of 1000 triads

Number of SNPs	Number of chromosomes	Disease prevalence or QT	Time used (seconds)
10,279	4	0.0002	159
10,279	4	0.00166	104
10,279	4	0.01	94
10,279	4	0.1	89
566,393	22	0.0002	2258
566,393	22	0.00166	2268
566,393	22	0.01	2190
566,393	22	0.1	2252
10,279	4	QT	92
566,393	22	QT	2223

## Discussion

The principal novelty to our resampling approach is our use of complement triads and our use of sets of chromosomal fragments from the triple of mother-father-child genotypes as the re-sampling unit. The inclusion of all of the complement triads effectively destroys signal from the original GWAS, as was demonstrated. Our resampling procedure, before any assignment of SNP-associated traits, recapitulates the allele frequencies in the case-parents input data rather than those in the underlying source population. The two can differ because any allele (including interacting alleles) that is positively associated with the offspring phenotype will have a slightly higher prevalence in the parental genotypes of the observed triads than in the source population from which they came; that enrichment will be propagated to the simulated triads. We selected three break points per chromosome in our simulations for convenience. Some researchers may prefer to take the chromosome size into consideration when picking the number of break points, and our R functions allow users to specify the number of breakpoints separately for each chromosome. The idea of using chromosomal fragments broken at recombination hotspots as part of a resampling scheme has been employed by others. It was used to simulate case-control data [9] and to increase diversity through simulated crossover [7]. Our approach, which also uses a newly chosen set of breakpoints for each simulated triad, creates simulated data with genetic diversity while retaining the realistic LD structure from the original data. It also foregoes the random mating assumptions inherent in many genetic simulators.

Our framework is more broadly applicable than our current software implementation supports. In addition to the study designs mentioned, one could simulate data based on outcome-dependent (extreme phenotype) sampling for a quantitative trait: after simulating offspring-parent data for the quantitative trait, the probability of inclusion into the simulation sample would depend on a user-specified function of the trait value. Additional risk models could be incorporated. For example, instead of the present multiplicative structure in eq. (1), one could build an additive structure. Risk models could allow for the effects of maternal genes acting during pregnancy on offspring phenotype as well as maternal exposures and parent-of-origin effects. Maternal-fetal genotype interactions could also be simulated. Dichotomous traits could be extended to polytomous traits and univariate quantitative traits to multivariate quantitative traits. Error distributions other than the normal errors of eq. (2) could be incorporated. An important extension would be to accommodate a richer genetic structure for each pathway. Currently, our code is restricted to a dominant (at least one variant present) mode of inheritance for each SNP in a pathway; our framework would allow more flexibility in that specification, ideally the Boolean specifications used in logic regression [10, 11].

Our approach does have some inherent limitations. Any re-sampling-based approach such as ours may be limited to an extent by the original data. Ideally the triads to be used as the raw material should include a large set of unrelated families. Also, to simulate stratified populations with our approach, the available data needs to contain distinct sub-populations. Also, unlike simulations based on mimicking evolutionary processes, resampling approaches cannot introduce new variants into a simulated population. For many purposes, this drawback is minor though it may be relevant when studying rare variants. Our approach may not be ideal for simulating rare variants. SeqSIMLA [12], a coalescent-based simulator for either unrelated case-control or family samples, and RarePedSim [13], a forward-time simulator for general pedigree structures, are two packages designed specifically to simulate sequence-based data incorporating rare variants into the determination of phenotypes. Because our approach simulates a genotype at each locus, it cannot provide simulated haplotypes. If haplotypes are needed, a web-based tool HAP-SAMPLE is available that relies on resampling chromosome-length haplotypes derived from 30 triads in the HapMap project [7] and can simulate both case-control and case-parents data. This tool has some restrictions, however, that make it unattractive compared to our approach when only genotypes are of interest. HAP-SAMPLE assumes random mating and can include at most one risk locus per chromosome; neither restriction applies to our approach. In addition, the small original sample of chromosome-length haplotypes currently available to HAP-SAMPLE would tend to limit the genetic diversity available in any simulated data sets versus that achievable with the larger number of case-parents GWAS studies that could be used by our approach.

As always, the choice of a simulation method will depend on the goals of the project. If assessment of methods for studying genome-wide genetic associations, particularly those involving multi-SNP epistasis, is the goal, our method could serve this purpose well.

## Conclusion

We have provided a resampling-based method to simulate autosomal SNP genotypes for use in evaluating data-analysis methods. The required raw-materials input for these simulations is GWAS triad genotype data from individuals and their parents. Our approach can simulate both case triads but also control triads and offspring with quantitative traits (with or without their parents). Discarding parents from case triads provides case-only samples and discarding parents from both case and control triads provides case-control samples. We showed through simulations that our method produces simulated data sets that largely preserve the allele frequencies and the realistic SNP-pair LD structure that existed in the original data. Using our approach, one can simulate complex scenarios that involve multiple genetic pathways, each containing multiple interacting SNPs, pathways that possibly interact with dichotomous environmental factors.

### Availability and requirements

Project name: TriadSim.

Project home page: https://cran.r-project.org/web/packages/TriadSim/index.html

Operating system: Platform independent.

Programming language: RLicense: GPL-3.

## Additional file

Additional file 1: Fig. S1. Genotype correlation (R) between rare SNP pairs within 200Kb of each other in the original data plotted against the corresponding R in a single simulated data set. Red triangles represent the SNP pairs with an observed R that differs from that based on the original data by at least 0.1 (LD discrepant pairs). a) 0% discrepant among 16 pairs of SNPs both with 0.04 < MAF ≤ 0.05 in the original data; b) 0% discrepant among 26 pairs of SNPs both with 0.03 < MAF ≤ 0.04; c) 2.6% discrepant among 38 pairs of SNPs both with 0.02 < MAF ≤ 0.03; d) 8.6% discrepant among 35 pairs of SNPs both with 0.01 < MAF ≤ 0.02; e) 31% discrepant among 13 pairs of SNPs both with 0.005 < MAF ≤ 0.01; f) 14.2% discrepant among 296 pairs of SNPs both with MAF ≤ 0.005. **Fig. S2.** Average squared genotype correlations (R^2^) between loci plotted against the distance between them. This figure is similar to Fig. 2 in the text but instead it shows the LD decay for SNPs up to 200 kbps apart (to facilitate comparison to Additional file 1: Fig. S3). The black line shows the curve based on the original data while the red line shows the corresponding averaged value based on 1000 simulated data sets. The two lines coincide and only the red line is visible. **Fig. S3.** Average squared genotype correlations (R^2^) between loci plotted against the distance between them for rare SNPs. The black line shows the curve based on the original data while the red line shows the corresponding averaged value based on 1000 simulated data sets. When the two lines coincide only the red line is visible. a) 1782 pairs of SNPs both with MAF ≤ 0.05; b) 1495 pairs of SNPs both with MAF ≤ 0.04; c) 1147 pairs of SNPs both with MAF ≤ 0.03; d) 848 pairs of SNPs both with MAF ≤ 0.02; e) 593 pairs of SNPs both with MAF ≤ 0.01; f) 446 pairs of SNPs both with MAF ≤ 0.005. **Fig. S4** Comparison of minor allele frequencies (MAFs) in the original data versus those in a single simulated data set for rare SNPs (MAF ≤ 0.05). The crosses represent the SNPs with MAF in the simulated data that fall outside 95% binomial prediction intervals calculated using the MAF in the original data as the true MAF (these MAF discrepant SNPs should make up about 5% of SNPS by definition). The colors denote SNPs in different MAF ranges in the original data: *orange*, 2.8% discrepant among 178 SNPs with 0.04 < MAF ≤ 0.05; *blue*, 5.6% discrepant among 214 SNPs with MAF 0.03 < MAF ≤ 0.04; *green*, 4.8% discrepant among 228 SNPs with 0.02 < MAF ≤ 0.03; *purple*, 5.2% discrepant among 248 SNPs with 0.01 < MAF ≤ 0.02; *red*, 7.9% discrepant among 151 SNPs with 0.005 < MAF ≤ 0.01; *black*, 4.7% discrepant among 852 SNPs with MAF ≤ 0.005. Overall, 4.97% of 1871 SNPs with MAF ≤ 0.05 lay outside their corresponding 95% prediction interval. **Fig. S5** Empirical coverage of nominal 95% binomial prediction intervals for rare SNPs (MAF ≤ 0.05) plotted against the SNP’s minor allele frequency (MAF) in the original data. Prediction intervals are calculated for each SNP in each simulated data set using the SNP’s MAF in the original data as its true MAF. Empirical coverage for a SNP is calculated as the proportion of 1000 simulated data sets in which the SNP’s observed MAF was within its prediction interval. Each point represents empirical coverage for one of 1871 SNPs with MAF ≤ 0.05 in the simulations, based on 1000 simulated data sets. The horizontal reference lines correspond to mean and median coverage across all 10,279 SNPs in the simulations (both 95%, matching the nominal coverage) and to the 2.5th and 97.5th percentiles (93% and 97%, respectively). (DOCX 1187 kb)

## Abbreviations

GWAS: Genomewide association studies
LD: Linkage disequilibrium
MAF: Minor allele frequency
OR: Odds ratio
RR: Relative risk
SNP: Single nucleotide polymorphism
