# Supplementary material for: Simulating autosomal genotypes with realistic linkage disequilibrium and a spiked-in genetic effect
Source: BMC Bioinformatics. 2018 Jan 2;19:2. doi: 10.1186/s12859-017-2004-2 (PMC5749028; doi:10.1186/s12859-017-2004-2)

Additional files

**Additional file 1: Figure S1.** Correlation (R) between rare SNP pairs within 200Kb of each other in the original data plotted against the corresponding R in a single simulated data set. Red triangles represent the SNP pairs with an observed R that differs from that based on the original data by at least 0.1 (LD discrepant pairs). a) 0% discrepant among 16 pairs of SNPs both with 0.04 < MAF ≤ 0.05 in the original data; b) 0% discrepant among 26 pairs of SNPs both with 0.03 < MAF ≤ 0.04; c) 2.6% discrepant among 38 pairs of SNPs both with 0.02 < MAF ≤ 0.03; d) 8.6% discrepant among 35 pairs of SNPs both with 0.01 < MAF ≤ 0.02; e) 31% discrepant among 13 pairs of SNPs both with 0.005 < MAF ≤ 0.01; f) 14.2% discrepant among 296 pairs of SNPs both with MAF ≤ 0.005.

**Additional file 1: Figure S2.** Average squared correlations (R^2^) between SNPs plotted against the distance between them. This figure is similar to Figure 2 in the text but instead it shows the LD decay for SNPs up to 200 kbps apart (to facilitate comparison to Supplemental Figure 3). The black line shows the curve based on the original data while the red line shows the corresponding averaged value based on 1000 simulated data sets. The two lines coincide and only the red line is visible.

**Additional file 1: Figure S3.** Average squared correlations (R^2^) between SNPs plotted against the distance between them for rare SNPs. The black line shows the curve based on the original data while the red line shows the corresponding averaged value based on 1000 simulated data sets. When the two lines coincide only the red line is visible. a) 1782 pairs of SNPs both with MAF ≤ 0.05; b) 1495 pairs of SNPs both with MAF ≤ 0.04; c) 1147 pairs of SNPs both with MAF ≤ 0.03; d) 848 pairs of SNPs both with MAF ≤ 0.02; e) 593 pairs of SNPs both with MAF ≤ 0.01; f) 446 pairs of SNPs both with MAF ≤ 0.005.

**Additional file 1: Figure S4.** Comparison of minor allele frequencies (MAFs) in the original data versus those in a single simulated data set for rare SNPs (MAF≤ 0.05). The crosses represent the SNPs with MAF in the simulated data that fall outside 95% binomial prediction intervals calculated using the MAF in the original data as the true MAF (these MAF discrepant SNPs should make up about 5% of SNPS by definition). The colors denote SNPs in different MAF ranges in the original data: *orange*, 2.8% discrepant among 178 SNPs with 0.04 < MAF ≤ 0.05; *blue*, 5.6% discrepant among 214 SNPs with MAF 0.03 < MAF ≤ 0.04; *green*, 4.8% discrepant among 228 SNPs with 0.02 < MAF ≤ 0.03; *purple*, 5.2% discrepant among 248 SNPs with 0.01 < MAF ≤ 0.02; *red*, 7.9% discrepant among 151 SNPs with 0.005 < MAF ≤ 0.01; *black*, 4.7% discrepant among 852 SNPs with MAF ≤ 0.005. Overall, 4.97% of 1871 SNPs with MAF≤ 0.05 lay outside their corresponding 95% prediction interval.

**Additional file 1: Figure S5.** Empirical coverage of nominal 95% binomial prediction intervals for rare SNPs (MAF≤ 0.05) plotted against the SNP’s minor allele frequency (MAF) in the original data. Prediction intervals are calculated for each SNP in each simulated data set using the SNP’s MAF in the original data as its true MAF. Empirical coverage for a SNP is calculated as the proportion of 1000 simulated data sets in which the SNP’s observed MAF was within its prediction interval. Each point represents empirical coverage for one of 1871 SNPs with MAF≤ 0.05 in the simulations, based on 1000 simulated data sets. The horizontal reference lines correspond to mean and median coverage across all 10,279 SNPs in the simulations (both 95%, matching the nominal coverage) and to the 2.5^th^ and 97.5^th^ percentiles (93% and 97%, respectively).

**Additional file 1:** Figure S1


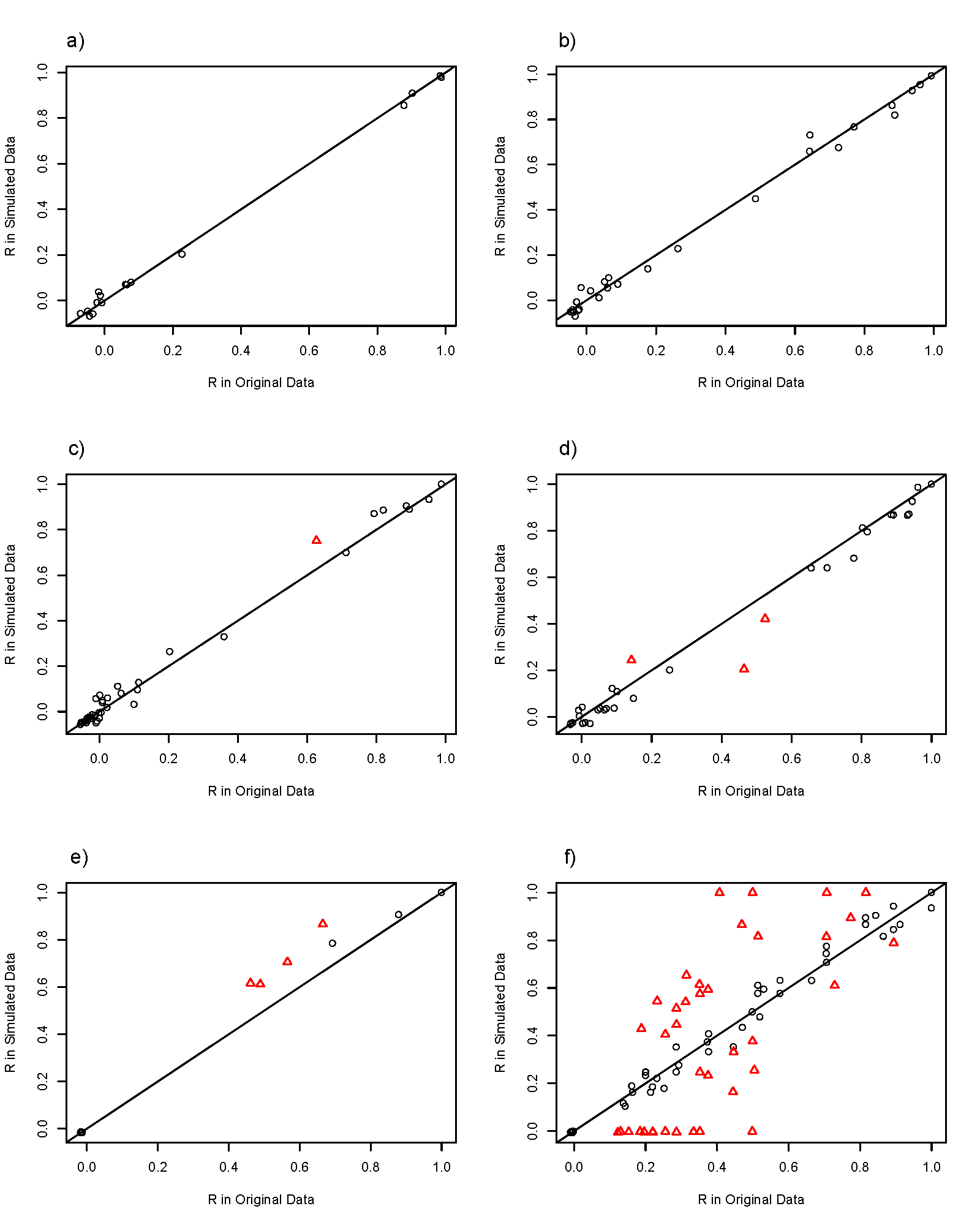


**Additional file 1:** Figure S2


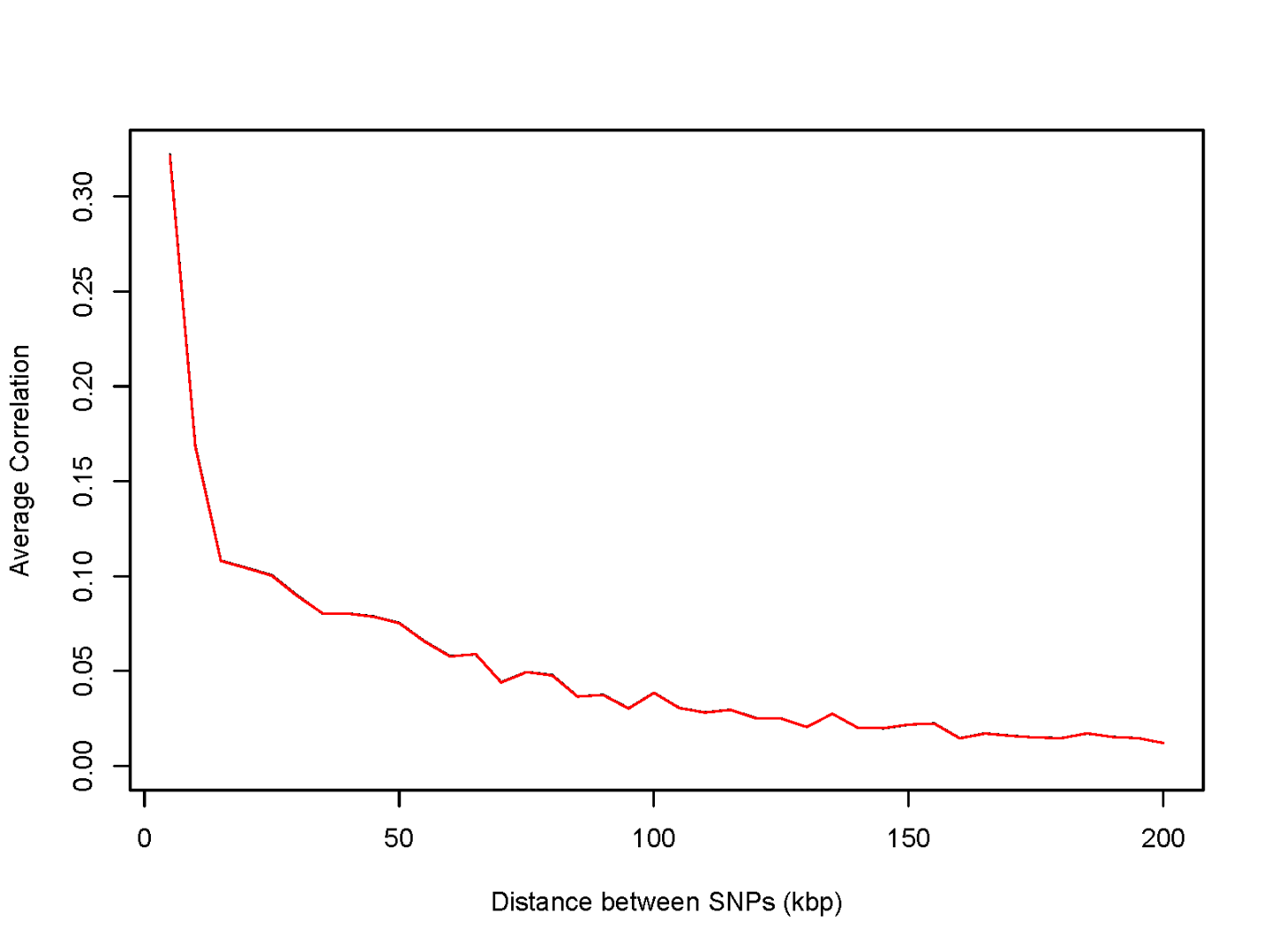


**Additional file 1:** Figure S3


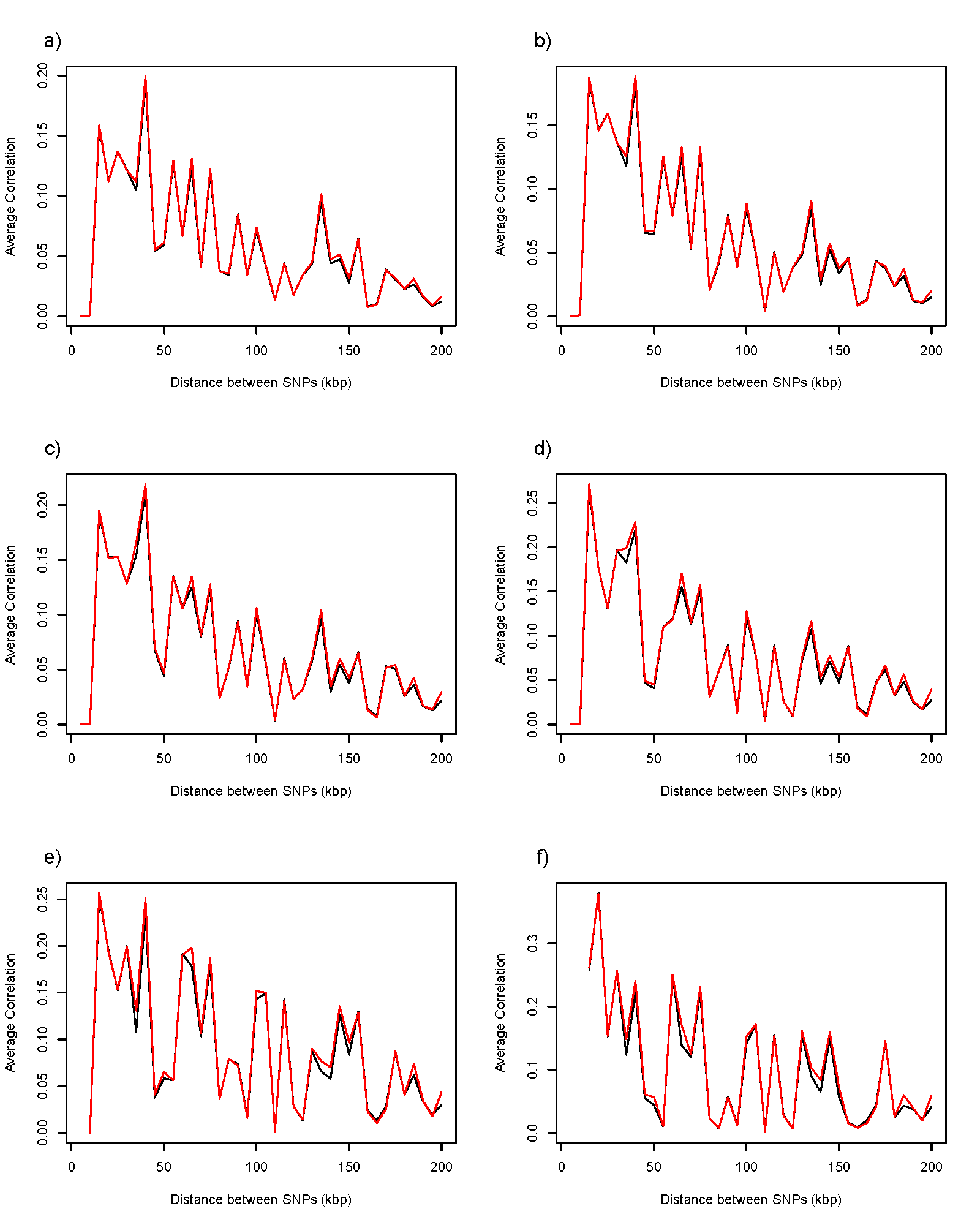


**Additional file 1:** Figure S4


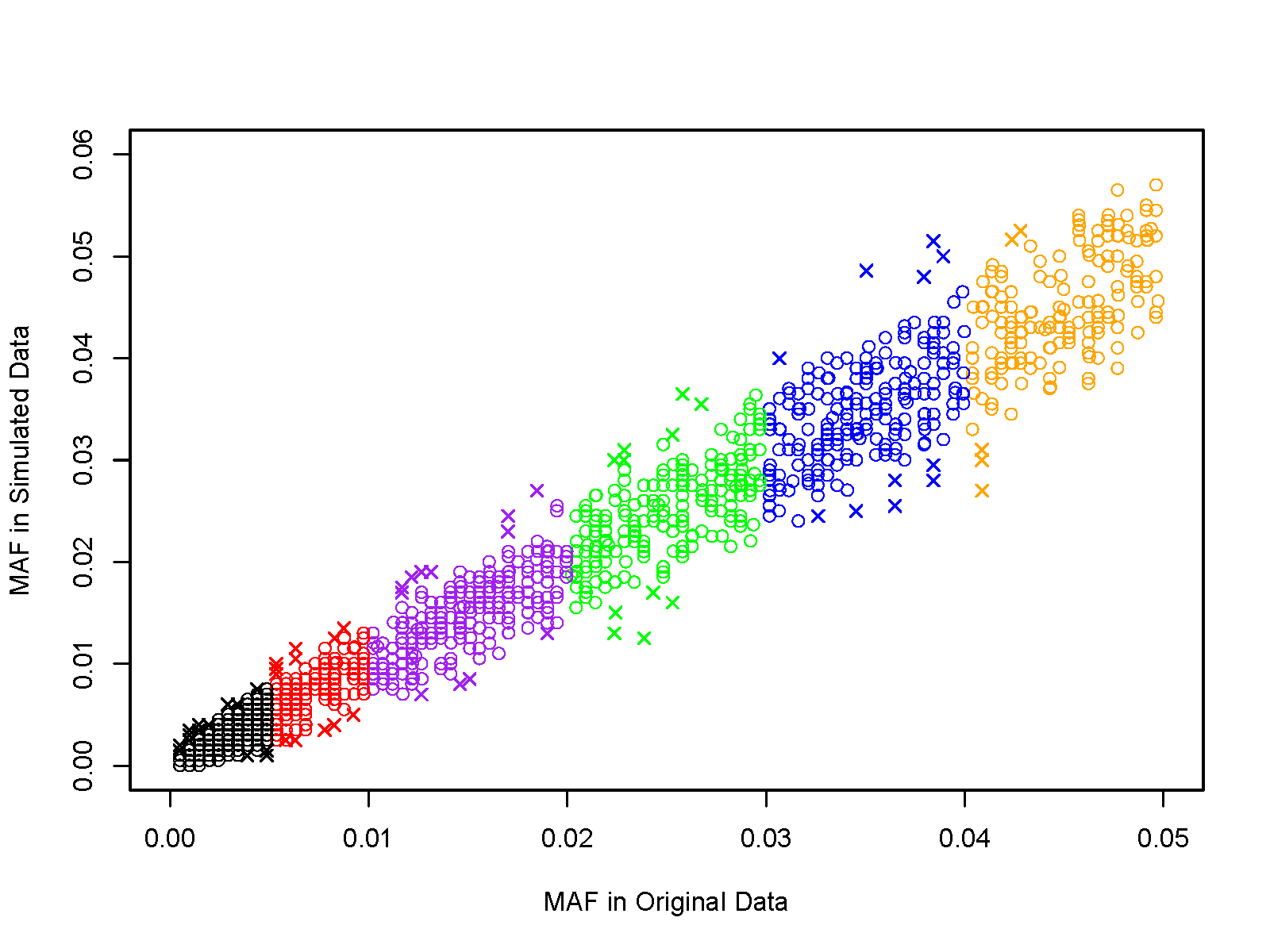


**Additional file 1:** Figure S5.


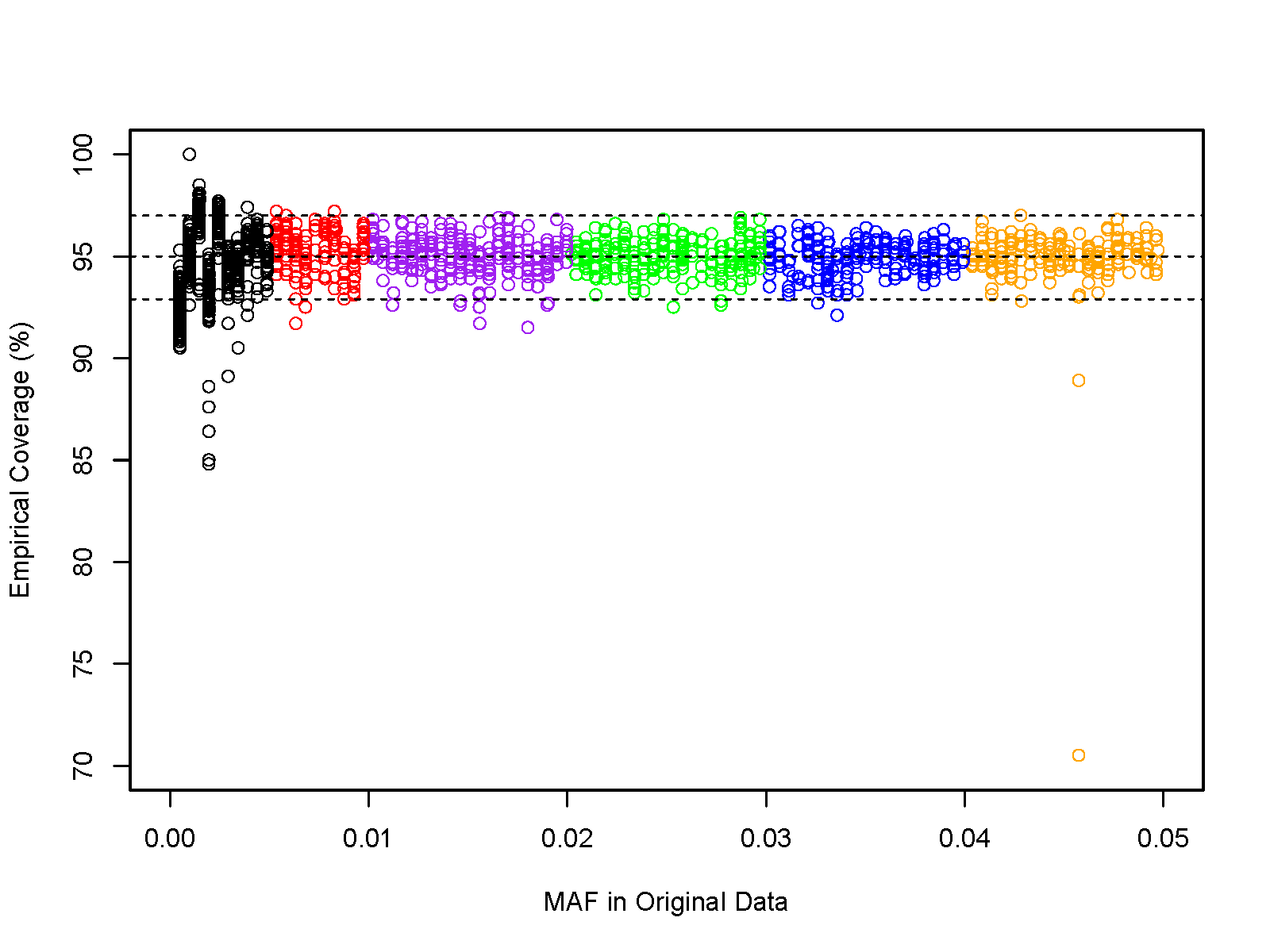

Supplement: Additional file 1: Fig. S1. — Genotype correlation (R) between rare SNP pairs within 200Kb of each other in the original data plotted against the corresponding R in a single simulated data set. Red triangles represent the SNP pairs with an observed R that differs from that based on the original data by at least 0.1 (LD discrepant pairs). a) 0% discrepant among 16 pairs of SNPs both with 0.04 < MAF ≤ 0.05 in the original data; b) 0% discrepant among 26 pairs of SNPs both with 0.03 < MAF ≤ 0.04; c) 2.6% discrepant among 38 pairs of SNPs both with 0.02 < MAF ≤ 0.03; d) 8.6% discrepant among 35 pairs of SNPs both with 0.01 < MAF ≤ 0.02; e) 31% discrepant among 13 pairs of SNPs both with 0.005 < MAF ≤ 0.01; f) 14.2% discrepant among 296 pairs of SNPs both with MAF ≤ 0.005. Fig. S2. Average squared genotype correlations (R2) between loci plotted against the distance between them. This figure is similar to Fig. 2 in the text but instead it shows the LD decay for SNPs up to 200 kbps apart (to facilitate comparison to Additional file 1: Fig. S3). The black line shows the curve based on the original data while the red line shows the corresponding averaged value based on 1000 simulated data sets. The two lines coincide and only the red line is visible. Fig. S3. Average squared genotype correlations (R2) between loci plotted against the distance between them for rare SNPs. The black line shows the curve based on the original data while the red line shows the corresponding averaged value based on 1000 simulated data sets. When the two lines coincide only the red line is visible. a) 1782 pairs of SNPs both with MAF ≤ 0.05; b) 1495 pairs of SNPs both with MAF ≤ 0.04; c) 1147 pairs of SNPs both with MAF ≤ 0.03; d) 848 pairs of SNPs both with MAF ≤ 0.02; e) 593 pairs of SNPs both with MAF ≤ 0.01; f) 446 pairs of SNPs both with MAF ≤ 0.005. Fig. S4 Comparison of minor allele frequencies (MAFs) in the original data versus those in a single simulated data set for rare SNPs (MAF ≤ 0.05). The crosses represent the SN [file 12859_2017_2004_MOESM1_ESM.docx]
